# Mother trees of common ash (*Fraxinus excelsior*) disperse different sets of mycobiome through their seed wings

**DOI:** 10.1186/s13104-024-06863-z

**Published:** 2024-07-30

**Authors:** Feng Long, James M. Doonan, Lene R. Nielsen, Erik D. Kjær, Chatchai Kosawang

**Affiliations:** Department of Geosciences and Natural Resource Management, University of Copenhagen, Rolighedsvej 23, 1958 Frederiksberg C, Denmark

**Keywords:** Seed mycobiome, Fraxinus excelsior, Common ash

## Abstract

**Objective:**

The endophytic mycobiome is present in all studied plant compartments, including fruits and seeds. Here, we studied the mycobiome of seed wings as they are transferred with seeds in common ash and tested whether the mycobiome differs among trees. To achieve this, we used ITS1-based amplicon sequencing and two genotypes of *F. excelsior* as a model to compare the mycobiome of mother trees and their wings.

**Results:**

We compared the mycobiome of 57 seed wings to the seed stalks (57) collected from two genotypes of *F. excelsior* using three ramets of each genotype. Alpha diversity indices (ACE, Fisher and Observed OTUs) suggested a higher richness of the mycobiome associated with the seed wing than the seed stalk within each genotype. However, there were neither significant differences in Shannon diversity between the mycobiomes from the two tissue types nor the two genotypes. PERMANOVA revealed significant differences in the mycobiome composition between tissue types (*P* < 0.001). It also showed a significant difference between seed wings (*P* = 0.04), but not between seed stalks of the two genotypes. Our results suggest that *Fraxinus excelsior* mother trees disperse different sets of mycobiomes with their seed wings, which may be important for germination and seedling establishment—especially in the light of ash dieback.

**Supplementary Information:**

The online version contains supplementary material available at 10.1186/s13104-024-06863-z.

## Introduction

The plant mycobiome is the collective group of fungi co-existing with plant hosts, which play a crucial role in growth and development of the host. The mycobiome is found ubiquitously in almost all studied plant compartments, including seeds and fruit [1–5]. Similar to other plant compartments, seed mycobiomes are both formed within tissues (endophytic mycobiome) and on the surfaces (epiphytic mycobiome) [5, 6]. Previous studies suggest that both vertical and horizontal factors contribute to the formation of seed mycobiomes [7, 8]. Despite limited research outside of model plant species, a growing body of evidence supports the beneficial nature of certain fruit-associated fungi in which they directly or indirectly benefit diverse physiological processes of the hosts [9, 10]. These processes include enhancing seed germination and promoting seedling vigor by fungal endophytes [11]. In addition, plant-associated fungi also can increase seed stress tolerance and contribute to pathogen resistance [12]. Recent evidence demonstrating that the seed mycobiome can affect successive microbial recruitment of seedlings [9] has underlined the need to better understand the composition and function of the seed-associated mycobiome.

Common ash (*Fraxinus excelsior*) is a fast-growing broadleaved species distributed across most of Europe [13, 14]. It has substantial ecological value as it grows in several forest types housing hundreds of associated organisms [14]. The dispersal unit of the species is the samara, which is the seed enclosed by a wing, and is developed from the ovary wall of the mother tree [15]. Previous attempt to study a transmission of the ash dieback causing fungal species, *Hymenoscyphus fraxineus*, through seeds of common ash identified a number of fungal taxa associated with the seeds [16]. However, the seed-associated fungal community is still not well understood. In this study, we used two *F. excelsior* genotypes to study the mycobiome of seed wings that are transferred with the seeds, and test whether these mycobiomes differ among trees. Mycobiome dispersal is particularly important to address in the light of the ongoing extensive common ash dieback (ADB) pandemic, caused by the invasive fungus, *Hymenoscyphus fraxineus*. The data we provide in this study contributes to a better understanding of the early dispersal of the mycobiome through seed wings in *F. excelsior* and provides a novel foundation for the conservation of the species.

## Materials and methods

### Plant materials

Samaras were collected from two *F. excelsior* mother trees (hereafter genotype 33 and 35) as these two genotypes were among a few mother trees that produced seeds with enough replications on 3rd of August 2021. The two genotypes were grown in a former Danish clonal seed orchard located at Tuse Næs, Zealand [17]. Genotype 35 was previously found to be superior to genotype 33 in terms of susceptibility to ash dieback (ADB) [17]. Eight to ten samaras were collected randomly from three different locations of each ramet, and three ramets were used per genotype. In total, 57 samaras from six mother trees (27 from genotype 33 and 30 from genotype 35) were used in this study. Samaras were surface sterilized as follows: 95% ethanol for 1 min, 3% sodium hypochlorite for 3 min, 95% ethanol for 1 min before being rinsed with sterile deionized water twice. Then samples were air dried in a laminar flow. Finally, we dissected part of the samara (seed wing) and seed stalk (Figure S1) from each samara in a laminar flow and kept the samples at − 20 °C for further processing.

### DNA manipulation and construction of ITS1 metabarcoding libraries

A fraction of the seed wing and seed stalk were homogenized at 30 Hz for 2 min twice using a RETSCH MM400 Mixer Mill (RETSCH, Germany). DNA was extracted using DNeasy PowerPlant Pro Kit (QIAGEN, Germany) following the manufacturer’s instruction and DNA concentration was determined using QUBIT3 fluorometer (ThermoFisher Scientific, USA). A two-step PCR approach was used to amplify fungal ITS1 region (208 ± 56 bp) and construct ITS1 amplicon libraries. PCR-I was performed in duplicate using five to ten nanograms of DNA, the primers BITS (5′-ACCTGCGGARGGATCA-3′) and B58S3 (5′-GAGATCCRTTGYTRAAAGTT-3′) [18] and Phusion Hot Start II High-Fidelity PCR Master Mix (ThermoFisher Scientific, Lithuania) on a BIO-RAD T100 thermal cycler (BIO-RAD, USA). A unique combinatorial Nextera XT v.2 barcode was introduced to each sample during PCR-II using the same polymerase as PCR-I, but the cycling number was limited to 12. The PCR-II products were purified using PureLink PCR Micro Kit (Invitrogen, USA), pooled in equimolar ratio, and sequenced with Illumina MiSeq platform with v3 chemistry (2 × 300 bases) at Macrogen Europe (Amsterdam, the Netherlands).

### Bioinformatics and statistical analyses

Only forward reads were used in this study. Low quality bases (Q < 20) and short reads (< 120 bases) were removed using BBduk in the BBtools suite v.38.90 (https://jgi.doe.gov/data-and-tools/software-tools/bbtools/). High quality reads were then parsed to QIIME2 version 2022.11 [19], where primers were removed with Cutadapt [20]. VSEARCH [21] was used for de novo OTU clustering at 99% identity and chimera identification. OTUs with frequency less than 0.005% of total reads were considered spurious [22] and removed from further analysis. Taxonomy assignment was performed using Sklearn [23] and the UNITE ITS database v.9 [24]. OTUs of interest were confirmed using BLASTn against nr/nt database of NCBI using the following cut-offs: e-value < e^−50^, pairwise identity > 99% and coverage > 90% [25]. All OTUs belonging to phyla other than fungi were removed from further analysis.

For diversity analyses, all samples were rarefied to 3031 reads per sample and used to calculate alpha (Observed OTUs, Abundance-based Coverage Estimator (ACE), Fisher and Shannon) and beta (Bray–Curtis) diversity indices within Phyloseq package [26]. A pairwise Kruskal–Wallis test was carried out to determine significant differences in the alpha indices between the two tissue types and the two genotypes. Bray–Curtis dissimilarity-based non-metric multidimensional scaling (NMDS) ordinations were plotted using Phyloseq [26] to explore variations between genotypes and tissue types. The Bray–Curtis index and permutational multivariate analysis of variance (PERMANOVA) with 1,000 permutations were used to compare mycobiome compositions from the two genotypes and two tissue types. All bioinformatic and statistical analyses were carried out within the QIIME2 space unless otherwise stated.

## Results and discussion

**Fig. 1:**
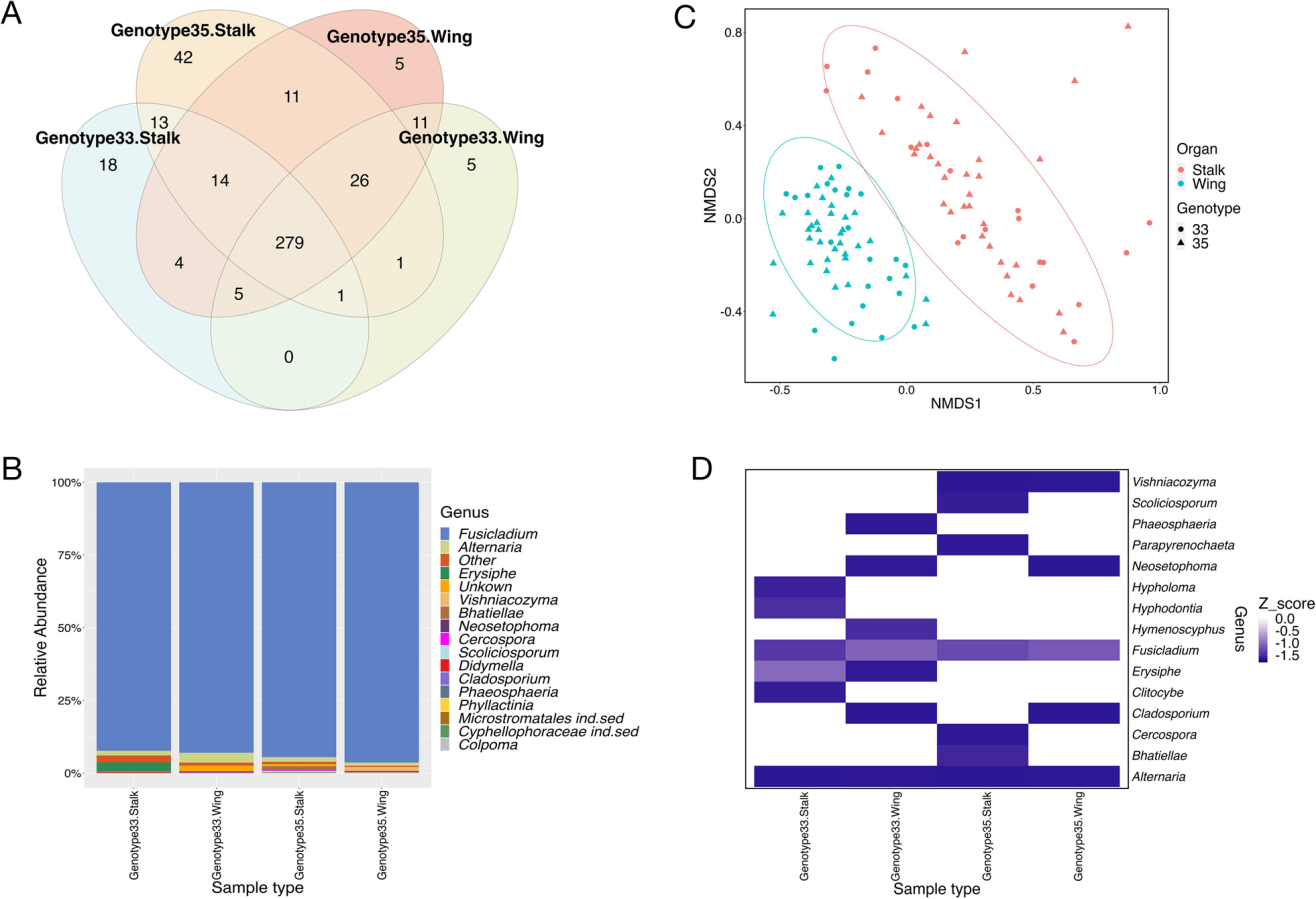
**A** Venn diagram showing OTUs between different common ash (*Fraxinus excelsior*) seed compartments and ash genotypes: **B** taxonomy bar plot of the fungal communities associated with seed stalk and seed wing of genotypes 33 and 35: **C** Non-multidimensional scaling (NDMS) based on Bray–Curtis dissimilarity of ash seed mycobiomes. The ellipses represent 95% confidence intervals for each tissue type: **D** heatmap illustrating the Z-score distribution of relative abundance of the top 15 dominant genera in seed samples

Our study generated a total of 333,410 high-quality reads from the two tissue types and two genotypes. Rarefaction curves were presented in Figure S2. Reads were subsequently clustered at 99% similarity giving a final set of 435 OTUs (Table S1). The majority of OTUs (365 OTUs; 84%) were present in both genotype 33 and genotype 35 (Fig. 1A), whereas 341 OTUs (78%) were shared between the two tissue types (Fig. 1A). Only a small fraction of OTUs were present uniquely either in the seed wing or seed stalk of each genotype. There was a higher number of OTUs in genotype 35 (412 OTUs) compared to genotype 33 (377 OTUs) (Fig. 1A) and a higher number of OTUs in seed stalk (414 OTUs) than in seed wing (362 OTUs) (Fig. 1A). This is in contrast with previous studies where the ADB-less susceptible genotype 35 was found to have lower species richness and species diversity of endophytic mycobiome compared to the more susceptible genotypes [27, 28]. However, those studies investigated the mycobiomes associated with other compartments i.e., twig and leaf, while the focus of this study was on the seed stalk and wing.

We were able to assign 413 out of 435 OTUs (94.9%) at genus level, leaving only 22 OTUs (5.1%) unidentified. The fungal communities associated with seed stalk and seed wings of genotype 33 and genotype 35 consisted of 59 genera. While the majority of OTUs were common to both genotypes, some were genotype-specific. For instance, 23 OTUs from 13 genera were specific to genotype 33, such as *Hypholoma*, *Alternaria* and *Clitocybe*, whereas 58 OTUs from 23 genera were found only in genotype 35 (Table S1), such as *Bhatiellae*, *Scoliciosporum* and *Parapyrenochaeta*. Irrespective of genotype, the communities were largely dominated by OTUs assigned to the genus *Fusicladium,* which occupied up to 94.3% of total reads and was present in both tissue types. Further annotation using BLASTn and the nr/nt database of NCBI identified these *Fusicladium* OTUs as *Fraxinicola fraxini*, a synonym of *Fusicladium proteae* [29]. Bilański and Kowalski (2022) reported *Venturia fraxini* (synonym of *F. fraxini*) as one of the most common endophytes detected in the asymptomatic petiole of common ash [30]. Despite its abundant presence, it did not exhibit an antagonistic effect on *H. fraxineus* [30]. In fact, *V. fraxini* can be a pathogen of *F. excelsior* [31–33] besides being an endophyte of *F. excelsior* [34]. With its high presence in seed wings, whether *V. fraxini* will form an endophytic relationship with the seedlings and whether it will enhance their growth and development merits consideration. Other less abundant genera included *Alternaria*, *Erysiphe* and *Vishniacozyma* (Fig. 1B, D). We also observed the presence of OTUs assigned to the ash dieback pathogen, *Hymenoscyphus fraxineus*, in the wing of the more susceptible genotype 33 while not in the less susceptible genotype 35 (Fig. 1D). The findings of *H. fraxineus* in some of the seed wings is comparable to previous findings of *H. fraxineus* found in seeds of the common ash collected from Latvia [16]. However, a following study from same research group specifically on ash seed embryos and seedlings did not detect any *H. fraxineus* [35]. Our finding supports that *H. fraxineus* can be transmitted by fruits or seeds, but we can neither validate its survival in the seeds nor its ability to cause disease in seedlings.

While the largest proportion of prevalent taxa were ascomycete fungi, a basidiomycete yeast from the genus *Vishniacozyma* was also present. One member of the genus *Vishniacozyma*, *V. victoriae* has previously been shown to be an antagonist of plant pathogens [36, 37]. In this study, OTUs annotated to *V. victoriae* were recorded more abundantly in the seed wing of genotype 35 than that of genotype 33. As genotype 35 is less susceptible to ADB than genotype 33, it is possible that the presence of *V. vitoriae* may be involved with ADB tolerance. Further investigation is needed to verify this hypothesis.

Fungi have different ecological roles, such as being pathotrophic and saprotrophic [38]. In our study, the identified genera were assigned to nine fungal guilds, including saprotrophs, plant pathogens, mycoparasites, epiphytes, and lichenized fungi. However, plant pathogens were largely dominant in common ash seeds (Figure S3). It is worth to note that these guilds were annotated using Fungaltrait, which is made up of a collection of fungal lifestyles from various plant hosts and studies. The OTUs assigned to the guild “pathogen” do not necessarily mean they are pathogens of *Fraxinus excelsior*.

**Fig. 2:**
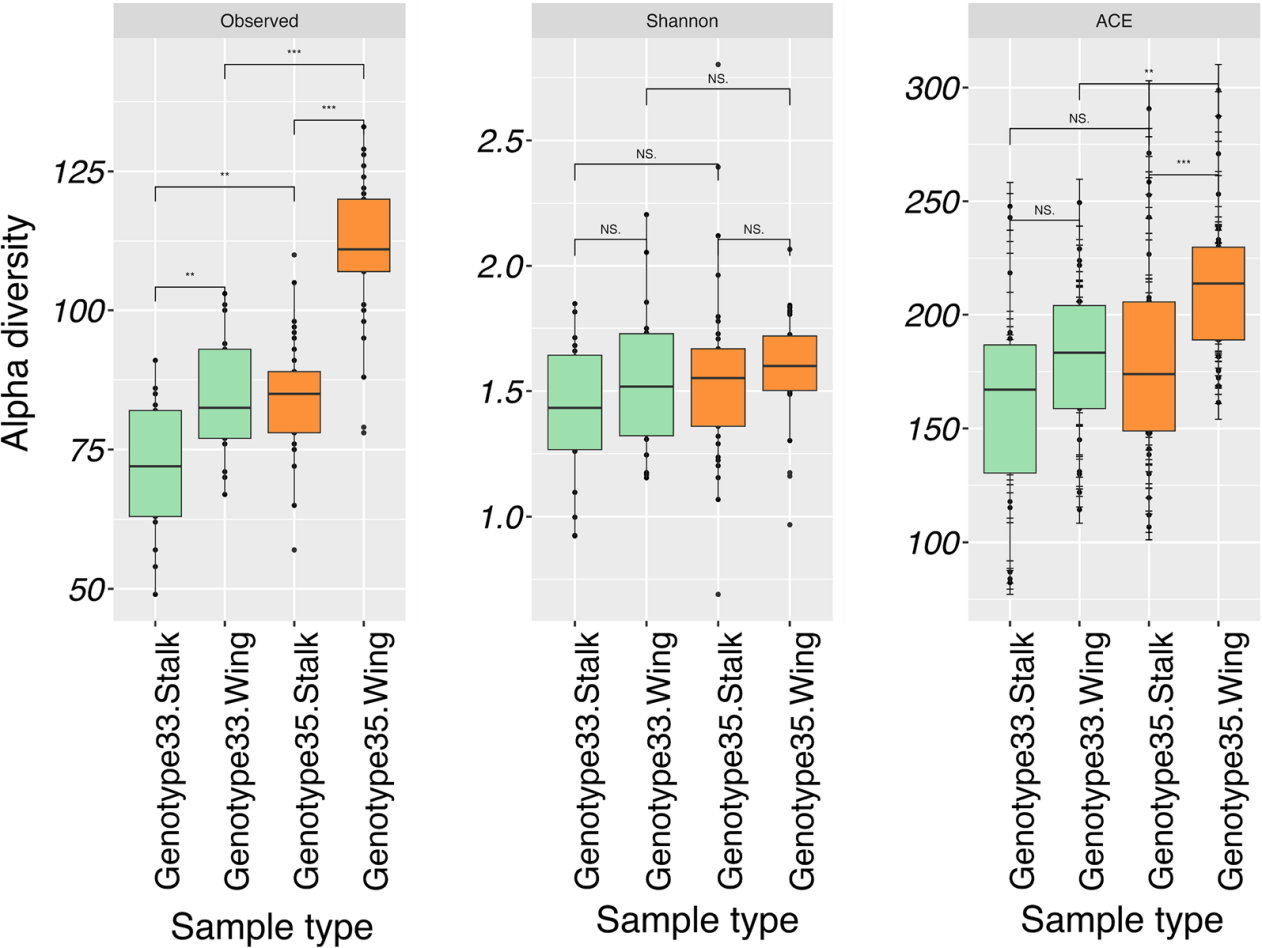
Box plot illustrating alpha diversity indices between tissue types and genotypes. *Denotes significance *p < 0.05, **p < 0.01 and ***p < 0.001

Diversity indices for species richness and other measures are presented in Fig. 2. The pairwise Kruskall-Wallis test identified significant differences in alpha diversity indices between the mycobiomes associated with seed wing of genotype 33 and 35 (ACE, P < 0.01; Fisher, *P* < 0.001; Observed OTUs < 0.001) and between the mycobiomes associated with seed stalk of genotype 33 and 35 (Observed OTUs, P < 0.01). However, we did not observe significant differences in Shannon diversity between the communities. The non-multidimensional scaling (NDMS) plot showed a clear separation between the fungal communities associated with different tissue types (Fig. 1C). Although separation between the two genotypes was less pronounced, PERMANOVA analysis based on the Bray–Curtis dissimilarity matrix revealed a significant difference between tissue types (*P* < 0.001) (Table S2). It also showed a significant difference between seed wing community compositions of genotype 33 and 35 (P < 0.05), but not between seed stalks of the two genotypes (Table S3). The results suggest differences in the fungal communities of the seed wing (the dispersal unit of seeds) and of different genotypes, but not between the communities of seed stalks.

## Conclusions

The mycobiome is increasingly recognized as a crucial component of plant health and ecosystem function. Understanding the composition and dynamics of the mycobiome related to seeds (here the seed wing) is central to the goal of advancing our understanding of plant–microbe interactions and developing sustainable agricultural/forest practices. Here we identified differences in mycobiome composition of seed wings collected from two different genotypes of *F. excelsior* with variations in susceptibility to ADB. These data suggest that the seed wing is a container carrying endophytes potentially important for growth and development of seeds from mother trees.

## Limitations

1. This experiment suffers from a relatively small sample size. While the number of seeds per genotype is adequate, the number of total genotypes is limited to two (clone 33 and clone 35). The small number of clones limits detailed statistical analyses e.g., identification of enriched fungal species.
2. Trees are home to fungi, bacteria and viruses. This study focuses only on stalk and seed-wing-associated fungal communities, bacterial and viral communities remain untouched. As this study does not reflect the entire seed microbiome, care should be taken when a connection between the seed microbiome and seed health is discussed.
3. Knowledge of fungal communities during seed germination is limited. The extent to which the seed mycobiome persists after vertical transmission is unknown. This study therefore requires follow-up experiments to further investigate the in situ roles of the mycobiome.

### Supplementary Information

Supplementary Material 1.

Supplementary Material 2.

Supplementary Material 3.

## Data Availability

All sequence data is available at NCBI under BioProject PRJDB17107.
