# Supplementary material for: Mother trees of common ash (Fraxinus excelsior) disperse different sets of mycobiome through their seed wings
Source: BMC Res Notes. 2024 Jul 30;17:213. doi: 10.1186/s13104-024-06863-z (PMC11289985; doi:10.1186/s13104-024-06863-z)
Supplement: Supplementary file 3 — Supplementary Material 3. [file 13104_2024_6863_MOESM3_ESM.pdf]

Supplementary Information

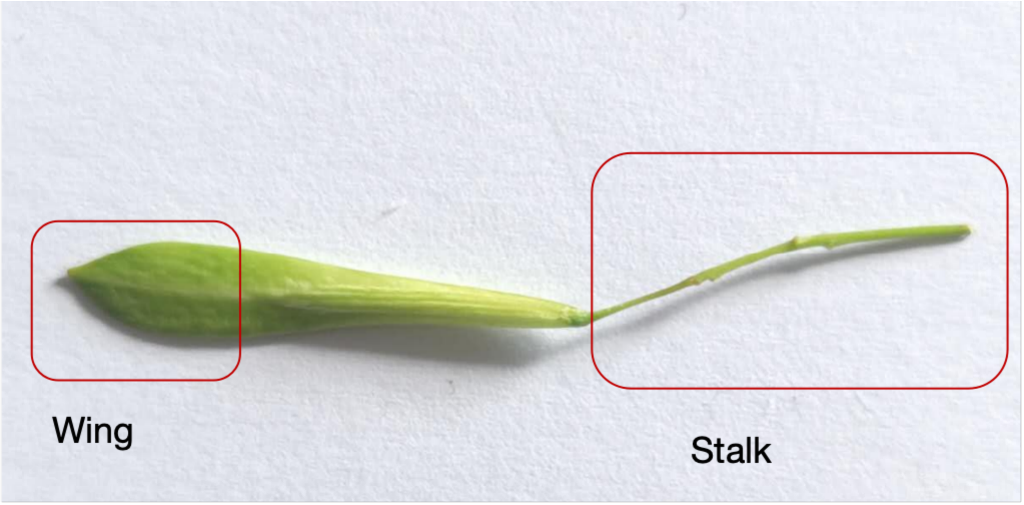

Figure S1. Tissue type overview including wing and stalk used for ITS1 sequencing

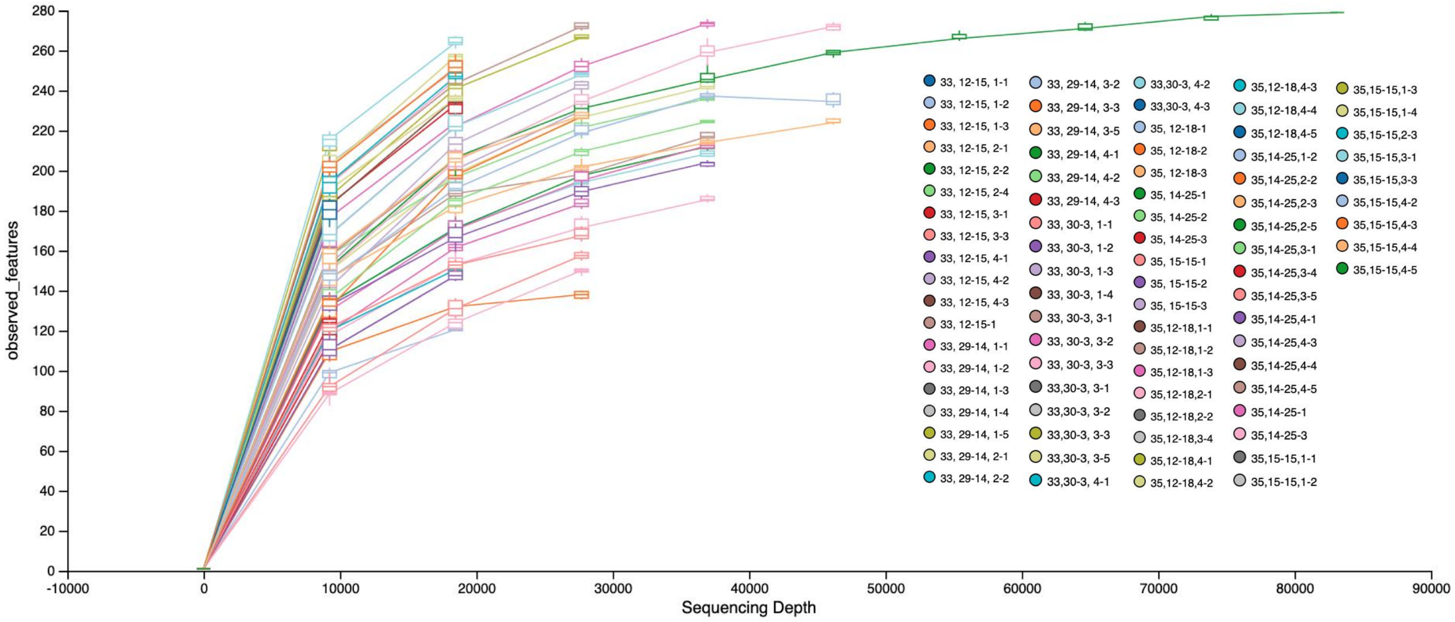

Figure S2. Rarefaction curve of each sample

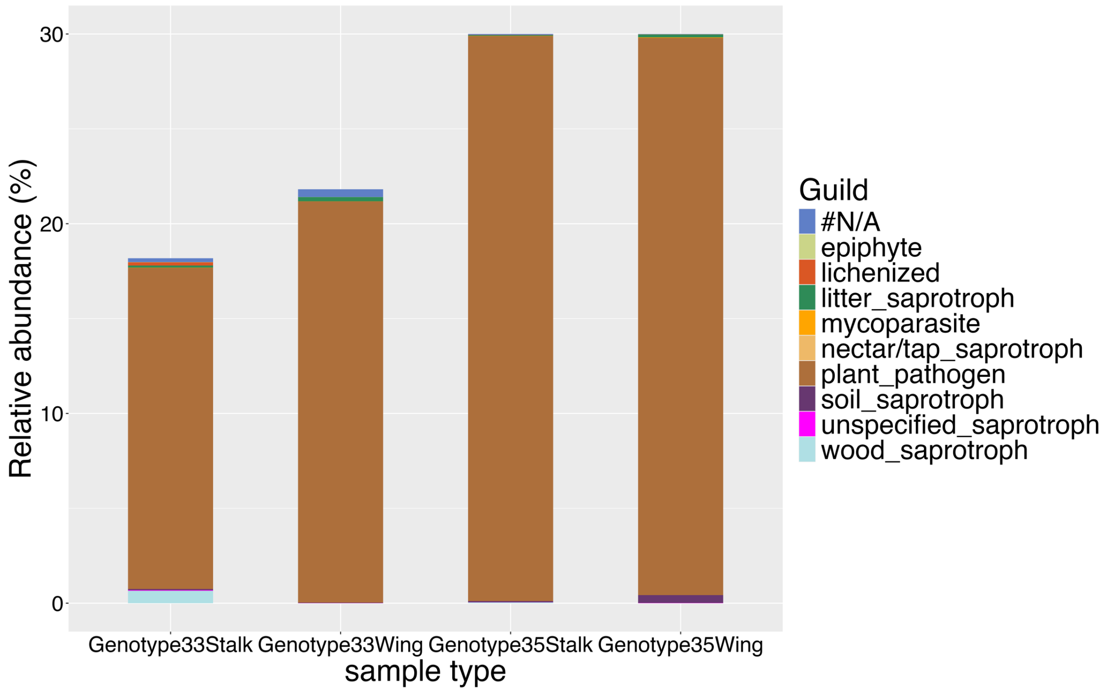

Figure S3. Relative abundance of the predicted guilds in different tissue types and samples. Please note that the assignment of guild is based on FungalTrait, which uses a collection of reports from different plants for guild assignment. OTUs predicted as “plant pathogen” are therefore not necessarily pathogens of common ash.
